# Relationship between body image and weight status in east Asian countries: comparison between South Korea and Taiwan

**DOI:** 10.1186/s12889-018-5738-5

**Published:** 2018-07-03

**Authors:** Jin-Won Noh, Young Dae Kwon, Youngmi Yang, Jooyoung Cheon, Jinseok Kim

**Affiliations:** 1 Department of Healthcare Management, Eulji University, Seongnam, Korea; 2 Global Health Unit, Department of Health Sciences, University Medical Centre Groningen, University of Groningen, Groningen, the Netherlands; 3 Department of Humanities and Social Medicine, College of Medicine and Catholic Institute for Healthcare Management, The Catholic University of Korea, Seoul, Korea; 4 Department of Social Welfare, Seoul Women’s University, Inmoonsawhoi-Kwan Bldg., 621 Hwarangno, Kongneung 2dong, Nowon, Seoul Korea; 5 Department of Nursing Science, Sungshin University, Seoul, Korea

**Keywords:** Body image, Weight status, Comparative study, East Asian countries

## Abstract

**Background:**

There are many studies examining the relationship between body image and weight status that compare Western and Asian countries. One limitation of these past studies was assuming that all Asian countries are a homogeneous group. To fill the gap in the literature, this study examined the relationship between body image and weight status between participants from two Asian countries.

**Methods:**

This study utilized data from the 2010 module of the East Asian Social Survey from South Korea (*n* = 1576) and Taiwan (*n* = 2199), which contained questions related to body image. Body image was originally measured using a five-point Likert-type question, which was collapsed into three categories for the analysis. Weight status was derived from body mass index scores, which were calculated using self-reported weight and height. A set of multinomial logistic regression analyses were used to investigate the relationship between body image and weight status, stratified by country.

**Results:**

A significant relationship between body image and weight status after controlling for relevant covariates was reaffirmed in this study in the South Korean and Taiwanese. Results indicated that the relationship between body image and weight status of the Taiwanese sample was similar to the relationship in the South Korean sample. However, the results from a further analysis showed that the strength of the relationship across the two Asian countries appeared to be different.

**Conclusions:**

The weight over-perception was more evident in South Korea than in Taiwan. Females were more vulnerable to societal pressures for thinness and the misperception of the ideal body than males. Interventions to improve distorted body image perception were needed in both countries.

## Background

Body image is a subjective image about one’s own body that plays a critical role in the perception of the physical self and the formation and representation of self-concept [1–3]. The body image can be divided into the ‘perceptual body image’, meaning an accurate perception or distorted awareness of one’s own body, and ‘attitudinal body image’, the attitude and feeling of one’s own body [2–7]. The perceptual body image today is known to be highly influenced by the media [2, 8–11]. Various body types and shapes portrayed by the media in television programs, movies, and magazines may shape viewers’ perceptions of ‘standard’ weight, overweight, and obese individuals [10, 12–14]. Specifically, overweight or obese people are often socially disparaged by the media because television in particular idealizes thin and slim characters [12].

On the other hand, attitudinal body image is closer to a feeling and attitude of the body. For instance, people that have been motivated by the thin and slim characters in the media and have exercised to keep their body fit tend to be more satisfied with their own bodies. However, most people are not satisfied with their body image after comparing their own bodies with slim characters portrayed in the media [5, 15–18].

Studies suggest that body image is associated with various psychological and emotional difficulties such as eating disorders, low self-esteem, depression, and anxiety [1, 7, 19–22]. Other studies of the psychological and emotional problems report that the perception that one has for one’s own body is similar to the perception that one has for oneself [20, 23].

It has been reported in the literature that the relationship between body image and actual weight status differs across various cultures and countries. In European countries, for example, overweight men and women tend to perceive themselves as normal weight, and obese men and women underestimate their weight status and categorize themselves as overweight [24]. Another study on people in the United States has shown that overweight Caucasian women tended to underestimate their weight, whereas overweight African American and Hispanic women did not report body image discrepancy [6]. In Asian studies, on the other hand, normal weight and underweight Taiwanese women had the tendency to view themselves as overweight, whereas Taiwanese men believe they are underweight [25, 26]. Korean women tend to overestimate their weight [27]. In Japan, similar to in Korea, underweight women view themselves as normal or overweight [28], and a high percentage of normal weight women believed themselves to be overweight [29]. In summary, many studies reported body image discrepancy, the pattern of which varies by different cultures and countries [30].

There are many studies examining the relationship between body image and weight status, but most of the studies have focused on samples from Western countries [6]. There are a few studies that compare Western and Asian countries in terms of the relationship between body image and weight status [31–33]. One limitation of these comparative studies was to assume that all Asian countries are a homogeneous group. There is a dearth of studies that compare multiple Asian countries in terms of BD. Using multi-national data from Asian countries, the present study aims to fill the aforementioned gap in the literature to compare the relationship between weight status and body image in samples from South Korea and Taiwan.

## Methods

### Data and subjects

This analysis utilized data from the East Asian Social Survey (EASS). The EASS was conducted through collaborative efforts in China, Japan, South Korea, and Taiwan. The EASS data was made available through Sungkyunkwan University of Seoul, Korea, which is in charge of the management and distribution of the data. The EASS was conducted biannually from 2006 and asked a module of questions with a different topic for each year of the survey. This study utilized the 2010 module of the EASS, which included health-related questions. Specifically, the data from South Korea and Taiwan were analyzed because only these two countries asked questions related to body image.

The sample population of the EASS in Taiwan and South Korea included men and women 18 years or older. A multi-stage stratified random sampling procedure was implemented for each country and the response rates were 49.7% in Taiwan and 63.0% in South Korea. Failed contact attempts were the biggest reason for no response (Korea: 16.8%; Taiwan: 24.5%), followed by refusal (Korea: 12.3%; Taiwan 18.9%) and other reasons (Korea: 7.9%; Taiwan: 3.3%). In the 2010 module of EASS data, there were 1576 participants from South Korea and 2199 from Taiwan. Finally, this study excluded 124 respondents who did not provide BMI-related data.

EASS data are accessible via a public database (http://www.eassda.org). Our study was performed in accordance with ethical standards and was reviewed and approved by the Institutional Review Board of Seoul Women’s University (SWU IRB-2017-6).

### Variables and measurement

Body image was measured using a question asking “What do you think about your body shape?” Respondents were asked to answer this question using a five-point Likert scale comprised of 1 = ‘a lot underweight’, 2 = ‘a little underweight’, 3 = ‘neither underweight nor overweight’, 4 = ‘a little overweight’, and 5 = ‘a lot overweight’. This study collapsed the first two responses into one category of ‘consider underweight’ and the last two responses into another category of ‘consider overweight’ in the analysis. BMI was defined as the body mass divided by the square of the height and was calculated in this study using the respondents’ self-reported weight and height. BMI scores were used to classify the participants as underweight (under 18.5 kg/m^2^), normal weight (between 18.5 kg/m^2^ and 23.0 kg/m^2^), overweight (between 23.0 kg/m^2^ and 25.0 kg/m^2^), obese (between 25.0 kg/m^2^ and 30.0 kg/m^2^), or severely obese (over 30.0 kg/m^2^) based on the World Health Organization’s classifications revised for the Asia-Pacific region. This study simplified the categories into three categories in the analysis: underweight/normal, overweight, and obese/severely obese. Other covariates in this analysis included age in years, gender (0 = male; 1 = female), marital status (1 = married or cohabiting; 0 = otherwise), region (1 = urban; 0 = otherwise), education in years, smoking behavior (1 = never smokes; 0 = otherwise) and alcohol consumption (1 = never drinks; 0 = otherwise).

### Statistical analysis

A set of descriptive analyses stratified by country were conducted to summarize the characteristics of the samples and compare the countries. Given the categorical nature of body image—the dependent variable—a set of multinomial logistic regressions was used to investigate the relationship between body image and BMI stratified by country. To test whether the relationship between body image and BMI was different across country, another regression model with cross product interaction terms between BMI and country was calculated. Stata MP 14 (StataCorp, College Station, TX, USA) was used for data management and analyses, and the threshold for the significance test was *p* < 0.05 (two-sided).

## Results

Characteristics of participants from Korea and Taiwan were summarized in Table 1. The proportion of people who were normal weight or underweight was higher in Korea (57.0%) than in Taiwan (47.2%) whereas the proportion of obese or severely obese participants was lower in Korea (22.8%) than in Taiwan (33.2%). People in Taiwan were more likely to view themselves as overweight (49.1%) and less likely to view themselves as normal weight (37.3%) than people in Korea (35.9 and 46.7%, respectively). These differences in weight status (Chi-squared (1) = 50.3, *p* < 0.001) and body image (Chi-squared (1) = 64.5, p < 0.001) in the two countries were significant. Gender distribution (Chi-squared (1) = 1.7, *p* = 0.187) and age (*t* = 1.6, *p* = 0.102) of the study participants between Korea and Taiwan were not significantly different. However, there were significant differences found in marital status (Chi-squared (1) = 7.6, *p* < 0.01), region (Chi-squared (1) = 9.4, *p* < 0.01), smoking status (Chi-squared (1) = 345.3, *p* < 0.001), alcohol drinking status (Chi-squared (1) = 5.2, *p* < 0.05), and years of education (*t* = 3.1, *p* < 0.01) (Table 1).

**Table 1:** Descriptive statistics

	Korea (*n* = 1576)		Taiwan (*n* = 2199)		Chi-squared
	N	(%)	N	(%)	
Weight status					50.29^***^
Underweight/Normal	887	57.0	988	47.2	
Overweight	314	20.2	412	19.7	
Obese/Severely obese	355	22.8	695	33.2	
Body shape image					64.49^***^
Underweight	273	17.4	299	13.6	
Normal	735	46.7	820	37.3	
Overweight	565	35.9	1078	49.1	
Gender: female	832	52.8	1113	50.6	1.74
Married (Cohabiting)	1007	64.1	1309	59.7	7.61^**^
Urban	1353	86.3	1802	82.6	9.37^**^
Never smoking	1136	72.3	1282	58.3	345.25^***^
Never drinking	1064	67.6	1562	71.1	5.21^*^
	Mean	SD	Mean	SD	t
Age (year)	48.94	58.5	46.78	17.7	1.63
Education year	11.89	4.3	11.40	5.1	3.12^**^

*SD* standard deviation

* *p* < 0.05, ** *p* < 0.01, *** *p* < 0.001

The distribution of weight status by gender for Korea and Taiwan, respectively, were summarized in Table 2. In Korea, there are 57.3% of female and 41.6% of male of normal weight, 11.3% of female and 2.7% of male of underweight, 14.3% of female and 26.7% of male of overweight, 15.4% of female and 26.2% of male of obese, and 1.8% of female and 2.8% of male of severely obese. In Taiwan, 8.1% of female and 3.5% of male were underweight, 48.4% of female and 34.6% of male normal weight, 16.8% of female and 22.2% of male overweight, 21.3% of female and 34.1% of male obese, and 5.4% of male and female, respectively, severely obese. Overall, these differences between male and female were significant in Korea (Chi-squared (4) = 112.3, *p* < 0.001) and in Taiwan (Chi-squared (4) = 82.9, *p* < 0.001) (Table 2).

**Table 2:** Distribution of weight status by gender

	South Korea			Taiwan		
	Male	Female	Total	Male	Female	Total
Underweight	20	92	112	37	84	121
	(2.7%)	(11.3%)	(7.2%)	(3.5%)	(8.1%)	(5.8%)
Normal	309	466	775	367	500	867
	(41.6%)	(57.3%)	(49.8%)	(34.6%)	(48.4%)	(41.4%)
Overweight	198	116	314	238	174	412
	(26.7%)	(14.3%)	(20.2%)	(22.2%)	(16.8%)	(19. 7%)
Obese	194	125	319	362	220	582
	(26.2%)	(15.4%)	(20.5%)	(34.1%)	(21.3%)	(27.8%)
Severely obese	21	15	36	57	56	113
	(2.8%)	(1.8%)	(2.3%)	(5.4%)	(5.4%)	(5.4%)
Total	742	814	1556	1061	1034	2095
	(100%)	(100%)	(100%)	(100%)	(100%)	(100%)
Chi-squared (4)	112.3^***^			82.9^***^		

* *p* < 0.05, ** *p* < 0.01, *** *p* < 0.001

As presented in Table 3, respondents’ self image of body shape tended to be coherent with their weight status both in Korea and in Taiwan. However, there were 35 Koreans (4.8%) and 38 Taiwanese (4.9%) who answered their body shape image being not underweight while they were underweight by their BMI. Also, there were 163 Koreans (47.1%) and 227 Taiwanese (32.8%) who consider themselves as overweight even though they were categorized as normal weight by their BMI (Table 3).

**Table 3:** Distribution of body image and weight status

		Body image						
	BMI weight status	A lot underweight	A little underweight	Neither underweight not overweight	A little overweight	A lot overweight	Total	Chi-squared(16)
South Korea	underweight	35	40	35	0	0	110	752.1^***^
		(48.6%)	(20.2%)	(4.8%)	(0%)	(0%)	(7.1%)	
	normal	35	146	431	149	14	775	
		(48.6%)	(73.7%)	(59.5%)	(32%)	(15.1%)	(49.9%)	
	overweight	0	10	176	112	15	313	
		(0%)	(5.1%)	(24.3%)	(24.1%)	(16.1%)	(20.2%)	
	obese	1	2	78	192	46	319	
		(1.4%)	(1%)	(10.8%)	(41.3%)	(49.5%)	(20.5%)	
	severely obese	1	0	5	12	18	36	
		(1.4%)	(0%)	(0.7%)	(2.6%)	(19.4%)	(2.3%)	
	total	72	198	725	465	93	1553	
		(100%)	(100%)	(100%)	(100%)	(100%)	(100%)	
Taiwan	underweight	28	55	33	5	0	121	1185.6^***^
		(38.9%)	(25.6%)	(4.3%)	(0.6%)	(0%)	(5.8%)	
	normal	42	136	462	213	14	867	
		(58.3%)	(63.3%)	(59.5%)	(27%)	(5.8%)	(41.4%)	
	overweight	2	17	167	202	24	412	
		(2.8%)	(7.9%)	(21.5%)	(25.6%)	(9.9%)	(19.7%)	
	obese	0	7	110	337	128	582	
		(0%)	(3.3%)	(14.2%)	(42.7%)	(52.9%)	(27.8%)	
	severely obese	0	0	4	33	76	113	
		(0%)	(0%)	(0.5%)	(4.2%)	(31.4%)	(5.4%)	
	total	72	215	776	790	242	2095	
		(100%)	(100%)	(100%)	(100%)	(100%)	(100%)	

* *p* < 0.05, ** *p* < 0.01, *** *p* < 0.001

Table 4 presents the results from multinomial logistic regression analysis of body image on weight status after controlling for other relevant covariates for Korea and Taiwan, respectively. The results showed that, in Korea, those who were overweight (B (SE) = − 2.66 (0.34), *p* < 0.001) or obese or severely obese (B (SE) = − 2.87 (0.52), *p* < 0.001) compared to underweight or normal weight, were less likely to consider themselves as underweight than as normal weight. Those who were overweight (B (SE) = 1.55 (0.19), *p* < 0.001) or obese or severely obese (B (SE) = 3.27 (0.21), *p* < 0.001) compared to underweight or normal weight were more likely to consider themselves as overweight than normal weight after controlling for relevant covariates such as age, gender, marital status, region, education, and health related behaviors such as smoking and alcohol drinking. Table 4 shows the same results for the Taiwan sample. After controlling for relevant covariates such as age, gender, marital status, region, education, and health related behaviors such as smoking and alcohol drinking, people in Taiwan who were overweight (B (SE) = − 1.70 (0.26), *p* < 0.001) or obese or severely obese (B (SE) = − 2.38 (0.41), *p* < 0.001) compared to underweight or normal weight, were less likely to consider themselves as underweight than as normal weight. Those who were overweight (B (SE) = 2.07 (0.17), *p* < 0.001) or obese or severely obese (B (SE) = 3.67 (0.18), *p* < 0.001), compared to underweight or normal weight, were more likely to consider themselves as overweight than normal weight in Taiwan (Table 4).

**Table 4:** Summary of the relationship between body image and weight status by country

	Korea								Taiwan							
	Underweight vs. Normal				Overweight vs. Normal				Underweight vs. Normal				Overweight vs. Normal			
	B		SE (B)	95% CI	B		SE (B)	95% CI	B		SE (B)	95% CI	B		SE (B)	95% CI
Underweight/Normal																
Overweight	−2.66	***	0.34	(−3.33, −1.99)	1.55	***	0.19	(1.18, 1.91)	−1.70	***	0.26	(−2.22, −1.18)	2.07	***	0.17	(1.75, 2.4)
Obese/Severely obese	−2.87	***	0.52	(−3.9, − 1.84)	3.27	***	0.21	(2.86, 3.68)	−2.38	***	0.41	(−3.18, −1.59)	3.67	***	0.18	(3.31, 4.02)
Age	0.00		0.00	(0, 0)	−0.01	*	0.01	(−0.02, 0)	0.00		0.01	(−0.01, 0.01)	−0.04	***	0.01	(−0.05, − 0.03)
Gender: female	− 0.81	***	0.18	(−1.17, − 0.44)	1.69	***	0.19	(1.31, 2.07)	−0.99	***	0.16	(−1.3, − 0.68)	1.57	***	0.14	(1.29, 1.85)
Married (Cohabiting)	0.05		0.16	(−0.27, 0.37)	0.03		0.15	(− 0.26, 0.32)	− 0.79	***	0.16	(−1.11, − 0.47)	0.14		0.13	(− 0.12, 0.41)
Urban	0.25		0.25	(− 0.23, 0.74)	− 0.12		0.21	(− 0.54, 0.3)	− 0.30		0.20	(− 0.69, 0.08)	0.36	*	0.16	(0.04, 0.68)
Education year	−0.01		0.02	(−0.05, 0.03)	0.05	*	0.02	(0.01, 0.09)	−0.02		0.02	(−0.06, 0.02)	0.07	***	0.02	(0.03, 0.1)
Never smoking	−0.67	***	0.19	(−1.04, −0.3)	0.06		0.18	(−0.3, 0.42)	0.08		0.18	(−0.28, 0.44)	0.05		0.15	(−0.25, 0.35)
Never drinking	0.31		0.18	(−0.04, 0.66)	−0.10		0.16	(−0.41, 0.21)	− 0.10		0.20	(− 0.5, 0.3)	−0.28		0.17	(−0.61, 0.05)

*SE* standard error, *CI* confidence interval

* *p* < 0.05, ** *p* < 0.01, *** *p* < 0.001

Results indicated that the Taiwanese sample showed a similar pattern as the Korean sample in the relationship between body image and weight status. Nevertheless, the strength of the relationship seemed to differ between the two Asian countries. We further tested whether the relationship between weight status and body image was different in Korea and Taiwan; results are summarized in Table 5. A multinomial logistic regression analysis results showed that the regression coefficient of the overweight group compared to the underweight or normal group in the likelihood of considering themselves as underweight than as normal weight was smaller in Taiwan compared to Korea (B (SE) = 0.84 (0.42), *p* = 0.046). In other words, while people both in Korea and Taiwan who were overweight were less likely to consider themselves as underweight than as normal weight, the relationship was stronger in the Korean sample compared to the Taiwanese sample (Table 5).

**Table 5:** Summary of the country effect on the relationship between body image and weight status

	Underweight vs. Normal				Overweight vs. Normal			
	B		SE (B)	95% CI	B		SE (B)	95% CI
Overweight*Taiwan	0.84	*	0.42	(0.01, 1.67)	0.32		0.21	(−0.1, 0.74)
Obese*Taiwan	0.37		0.65	(−0.91, 1.65)	0.11		0.22	(−0.32, 0.53)
Underweight/Normal								
Overweight	−2.59	***	0.34	(−3.25, −1.93)	1.65	***	0.17	(1.32, 1.99)
Obese/Severely obese	−2.78	***	0.52	(−3.8, −1.76)	3.41	***	0.19	(3.05, 3.78)
Taiwan	−0.22	+	0.12	(−0.45, 0.02)	0.41	**	0.14	(0.14, 0.67)
Age	0.00		0.00	(0, 0)	−0.02	***	0.00	(−0.03, − 0.02)
Gender: female	−0.94	***	0.12	(−1.16, −0.71)	1.64	***	0.11	(1.42, 1.86)
Married (Cohabiting)	−0.40	***	0.11	(−0.61, − 0.18)	0.04		0.10	(−0.15, 0.23)
Urban	−0.07		0.15	(−0.37, 0.23)	0.14		0.13	(−0.11, 0.39)
Education year	−0.01		0.01	(−0.03, 0.02)	0.06	***	0.01	(0.04, 0.09)
Never smoking	−0.30	*	0.13	(−0.54, − 0.05)	0.03		0.11	(−0.19, 0.24)
Never drinking	0.22	+	0.13	(−0.04, 0.47)	−0.14		0.11	(−0.36, 0.08)

* *p* < 0.05, ** *p* < 0.01, *** *p* < 0.001

*SE* standard error, *CI* confidence interval

## Discussion

Recent research has focused public attention on obesity and distorted body image, and found that cultural norms, ethnicity, and socioeconomic status influence the relationship between BMI and body image [34, 35]. According to the International Health and Behavior Survey, which collected data from 22 countries, perceptions of overweight in young men and women across BMI deciles were similar across diverse regions of the world, but Asians showed higher levels of trying to lose weight. Additionally, being female was related to feeling overweight at any BMI decile [35].

In this study, both Korean and Taiwanese individuals perceived they were overweight or obese even though they were not, which is consistent with previous studies [36–40]. As two neighboring East Asian countries, South Korea and Taiwan showed similar cultural and historical backgrounds, such as a Confucian culture, independence after World War II, rapid economic development, industrialization, and democratic politics [41]. In this study, Koreans were more overestimate their weight than Taiwanese to their body image. Body dissatisfaction, distorted perception of weight and body image, and disordered eating behaviors were more widespread in South Korea than in the West and China [42]. This weight over-perception and dissatisfaction of body image may explain the high rate of plastic surgery including liposuction in South Korea. According to statistics released by the International Society of Aesthetic Plastic Surgery [43], the estimated number of plastic surgeons was 2150 in South Korea, while 600 in Taiwan even though the population of South Korea is twice that of Taiwan [44, 45].

The rate of overestimation of their weight status among women was higher than that of men in both countries, but the discrepancy in BMI and body image in South Korea was stronger than in Taiwan in this study. Studies of Korean populations have consistently shown that Korean females perceived they are overweight or obese even though their weight was in the normal weight range [37, 38, 46, 47]. Korean females had a higher prevalence of weight over-perception than Korean males, which led to more weight control behavior [35, 37, 42], which may explained that disordered eating behaviors were more widespread in South Korea than in the West, China, and Japan [42]. This discrepancy in BMI and body image could be caused by their substantial exposure to mass media, which conveys the idea that losing weight is a high priority in females [48, 49]. This study could not use exposure to media or the effect of types of media on body image as a covariate, but many studies reported that exposure to the ‘thin ideal’ (the ideally slim female body) via television, magazine, film, social media, and advertisements made females perceived they were overweight [11, 18, 41]). Korean females showed high interest in their appearance and weight regardless of their actual body physique, and they in past research had concerns with weight and thin body shape, which led to reductions in both self-esteem and satisfaction with their appearance [47]. There are specific Korean terms to describe appearance thin body shape, such as ‘momjjang’ (a well-toned body) [50].

The desire to thin body shape may be related to inappropriate eating behavior and unhealthy weight loss behaviors in South Korea [51, 52]. A study compared the dietary pattern and body shape preference of female undergraduate students in South Korea and Japan [52]. Korean females showed a more irregular eating pattern and ate less frequently. Korean females’ ideal BMI was 18.4, which belonged to the underweight category [52]. In some severe cases, the misperception that they are obese even though they were not in Korean female could lead to eating disorders, such as anorexia and bulimia [53], depression, suicidal ideation, and suicidal plans [54].

There was no study to compare the perception of body image in South Korea to that of in Taiwan. Previous findings showed that body dissatisfaction was less common among males in Taiwan than those of the U.S. and Europe [55]. Taiwanese females had a weight over-perception compared to males much like females in South Korea. The ideal figure of females was thinner than the figure that males rated as most attractive to them, while the ideal figure of males was heavier than females actually preferred [40] Similar to in South Korea, media exposure acted as a conduit for thin-ideal messages, and led to increased body dissatisfaction, which ultimately contributed to restrained eating and unhealthy weight control behaviors [41].

This study has several limitations. The main limitation is that a cross-sectional study design cannot determine cause-effect relationships between BMI and self-rated body image. Another limitation is that this study cannot include all confounding covariates, which may have biased the study findings. Lastly, data from only two East Asian countries limits the generalizability of the study findings. In spite of several limitations, the study has some strengths. This study was the first study to compare the relationship between BMI and body image in South Korea to that in Taiwan, two neighboring countries with similar cultural and historical backgrounds. The findings provide insights in similarities and differences in weight over-perception in South Korea and Taiwan.

## Conclusion

This study examined the relationship between body image and weight status between participants from two East Asian countries. The relationship between body image and weight status of the Taiwanese samples was similar to the relationship in the South Korean samples. However, the results from a further analysis showed that the strength of the relationship across the two Asian countries appeared to be different. The weight over-perception was more evident in South Korea than in Taiwan. Females were more vulnerable to societal pressures for thinness and the misperception of the ideal body than males. Overall, interventions to amend distorted body image perception are needed in both countries.

## Abbreviations

BD: Body Image Discrepancy
BMI: Body Mass Index
EASS: East Asian Social Survey
